# Advances in meningioma genomics, proteomics, and epigenetics: insights into biomarker identification and targeted therapies

**DOI:** 10.18632/oncotarget.27841

**Published:** 2020-12-08

**Authors:** Ahmad A. Nazem, Jacob Ruzevick, Manuel J. Ferreira

**Affiliations:** ^1^Department of Neurosurgery, University of Washington School of Medicine, University of Washington Medical Center, Seattle, WA 98195, USA; ^*^These authors contributed equally to this work

**Keywords:** meningioma, skull base, NGS, sequencing, biomarker

## Abstract

Meningiomas are a heterogeneous group of tumors, defined histo-pathologically by World Health Organization (WHO) grading. The WHO grade of meningiomas does not always correlate with clinical aggressiveness. Despite maximal surgical resection and adjuvant radiation, a subset of tumors are clinically aggressive; displaying early recurrence and invasion. Current methods for identifying aggressive meningiomas solely focus on genomics, proteomics, or epigenetics and not a combination of all for developing a real-time clinical biomarker. Improved methods for the identification of these outlying tumors can facilitate better classification and potentially adjuvant treatment planning. Understanding the pathways of oncogenesis using multiple markers driving aggressive meningiomas can provide a foundation for targeted therapies, which currently do not exist.

## INTRODUCTION

Meningiomas are the most common central nervous system tumor, with an incidence of approximately 8 per 100/000 people [1]. They make up approximately one-third of all central nervous system tumors and can arise anywhere throughout the cranial-spinal axis. As a whole, meningiomas are a heterogeneous group of tumors and are currently graded according to the World Health Organization (WHO) classification, last revised in 2016. The WHO classifies meningiomas according to histological findings into one of three grades; WHO grade 1 (benign), WHO grade 2 (atypical), and WHO 3 (anaplastic) with fifteen histopathological subtypes. The histological characteristics leading to upgrading include 1) mitotic activity, 2) presence of brain invasion or, 3) at least three of five features including sheeting architecture, hypercellularity, necrosis, macronucleoli, or increased nuclear-to-cytoplasm ratio [2]. In addition, specific histological phenotypes, such as chordoid or clear cell morphologies are automatically grade 2 while rhabdoid and papillary morphologies are considered grade 3.

Despite the histological characteristics that define the WHO grade, clinical aggressiveness often does not correlate with WHO grading as up to 25% of grade 1 and grade 2 tumors exhibit rapid recurrence earlier than the estimated recurrence rate of 5–10% and 50% at 10 years respectively [3, 4]. Grade 3 meningiomas, the most aggressive of all meningiomas, recur after maximal surgical resection and adjuvant chemoradiation with an estimated median overall survival of 2.6 to 5.8 years with an overall 5-year survival ranging from 19–60% [5–9]. Across all tumors, histopathological grade and extent of surgical resection continue to provide the most widely accepted accuracy in prognosis. Regarding extent of resection, the Simpson grading scale, first reported in 1957, is the most often cited and classifies extent of resection into 5 grades which include 1) gross total resection including involved dura, 2) gross total resection with coagulation of affected dura, 3) gross total resection without removal or coagulation of affected dura, 4) subtotal resection or 5) biopsy only [10]. Rates of recurrence or progression correlate with increasing Simpson grade. While the initial 1957 study obviously did not stratify outcomes based on the current WHO classification, modern studies with relevant surgical techniques, instrumentation, and stratifying by WHO grade have re-examined the Simpson grading scale and continue to find it predictive for progression free survival [11, 12].

The current standard of care for meningiomas causing neurological symptoms due to compression or significant recurrence following a previously treated tumor is maximal safe surgical resection. This can be made challenging depending on tumor location and their proclivity to invade dural sinuses, encapsulate cranial nerves, and distort or encapsulate major cranial arteries. This is especially true for tumors arising from the skull base with this subset of tumors requiring extensive skull base removal for improved visualization and protection of adjacent brain and cranial nerves. WHO grade 1 tumors often show an indolent course and are often cured with surgery alone. Grade 2 tumors and rapidly recurring grade 1 tumors represent the most challenging tumors to longitudinally treat. A clinical benefit with adjuvant radiation, whether that be immediately post-operative or in a delayed fashion; has only been shown in non-randomized clinical series with some series showing no benefit [13–19]. Often, the decision regarding prescribing adjuvant radiation is based on age, health status, extent of resection, and institution-specific practice. Randomized trials attempting to elucidate the benefit of adjuvant radiation in Grade 2 meningiomas are actively enrolling. Grade 3 tumors require maximal surgical resection and adjuvant radiation. Salvage therapy often includes multiple repeat resections and chemotherapy, usually with limited success.

Refinements in histological evaluation and advancements in surgical techniques allowing for more radical resections for meningiomas has improved outcomes only minimally. Advancements in the genomic, proteomic, and epigenetic signature of meningiomas, though explosive in the last two decades have lagged in clinical translation behind other primary central nervous system tumors. In the case of gliomas, biomarkers such as isocitrate dehydrogenase (IDH) mutational status and O [6]-methylguanine DNA methyltransferase (MGMT) methylation are standard of care and provide prognostic insight into tumor behavior [20, 21]. These advancements in understanding the basic biology of glioma has allowed for a revolution in personalized therapy and should be used as an example of how understanding meningiomas at the genomic, proteomic, and epigenetic level can lead to improved therapies for a subset of patients with meningiomas that are at high risk for recurrence. Here, we review the current understanding of the meningioma genomic, proteomic, and epigenetic landscape, and discuss translational efforts to identify patients at high risk of recurrence and tumors with targetable mutations that may lead to more efficacious medical therapies.

### Genomic landscape in meningiomas

Advancements in the technology and the decreasing cost of next generation gene sequencing techniques has led to a notable advancement in the understanding of the genetic landscape leading to meningioma oncogenesis. The specific mutations involved in meningioma oncogenesis can be broadly categorized as tumors harboring NF2 mutations and tumors harboring mutations other than NF2.

**Figure 1:**
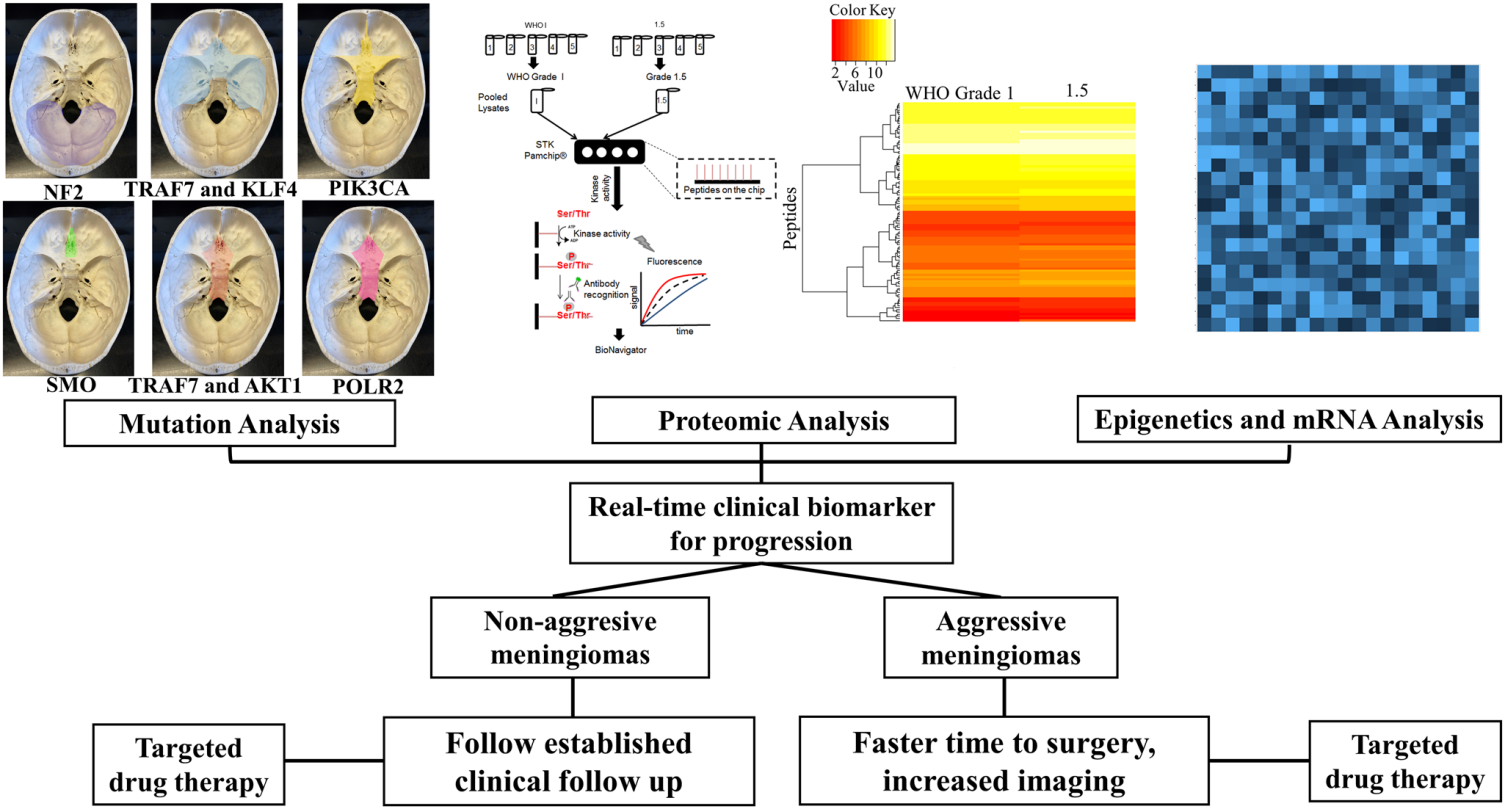
Multi-pronged approach for meningioma therapy. Using genomics, proteomics, epigenetics to develop a real-time clinical biomarker.

Approximately half of all sporadic meningiomas harbor an NF2 mutation with the remaining 50% of meningiomas harboring an alteration in one or a combination of several other genes involved in meningioma oncogenesis. These include tumor necrosis factor receptor associated factor 7 (TRAF7), alpha serine/threonine kinase 1 (AKT1), Krüppel-like factor 4 (KLF4), Smoothened (SMO), phosphatidylinositol-4,5-bisphosphate 3-kinase catalytic subunit alpha (PIK3CA), breast cancer type 1 susceptibility protein associated protein-1 (BAP1), polymerase RNA II polypeptide A (POLR2A), and phosphatidylinositol-4,5-biphosphaste 3-kinase catalytic subunit alpha chromatin remodeling gene (SMARCB1) [22–25]. While each gene variant has provided further information on the biology of meningioma; monosomy, NF2 and TRAF7 seem to be the driving forces in tumorigenesis. AKT1, KLF4, and SMO mutations rarely occur alone indicating that they might represent passenger mutations. Interestingly though, the location of intracranial and skull base meningiomas is highly correlative with each specific mutation (Figure 1) [22, 24, 26, 27]. Identifying mutations in BAP1, POLR2A and SMARCB1 and new genes might add to the understanding of these genomics subgroups.

Genomics provides a starting point to understand mechanistic signaling pathways involved in the early stages of tumorigenesis. Despite a revolution in the genomic landscape of meningiomas localizing to specific anatomical locations, alterations in high risk progression or recurrence tumors is lacking.

### NF2

The NF2 gene is a tumor suppressor gene present on chromosome 22q that encodes the protein, neurofibromin-2, a 69 kDa cytoskeleton scaffold protein. Neurofibromin-2 regulates the Hippo/SWH signaling pathway, resulting in tumor suppression by restricting proliferation and increasing apoptosis. In addition, neurofibromin-2 inhibits PI3 kinase by binding to AGAP2, impairing its stimulating activity, resulting in a link between two known oncogenic pathways [28]. Inactivation of neurofibromin-2 leads to the tumor syndrome neurofibromatosis type 2. This syndrome classically results in bilateral vestibular schwannomas, meningiomas, and ependymomas.

Approximately half of sporadic meningiomas of all grades are found to harbor NF2 mutations with an overwhelming majority having either complete or partial loss of heterozygosity on chromosome 22q leading to a nonfunctional neurofibromin-2. These can include frameshift, nonsense, or splice-site mutations [29]. Consistent with its role as a tumor suppressor, loss of function of neurofibromin-2 leads to upregulation of oncologic pathways, increased cellular proliferation, migration, and invasion, and decreased apoptosis. Patients that present with multiple meningiomas exhibit different NF2 mutations in respective tumors while identical biallelic NF2 mutations in patients with multiple tumors have been demonstrated, indicating that tumors can arise independently or through mosaicism or clonal spread [30]. When low- and high-grade tumors from a single patient were analyzed, NF2 loss was exhibited in both low and high grade tumors suggesting that NF2 loss is an early event in meningioma progression. This is further supported by recurrent tumors in the same patients exhibiting a 75% overlap of arm-level somatic copy number variations [31]. Tumors with NF2 mutations often localize to the cerebral convexity or posterior fossa skull base and have a limited representation in the midline skull base [24].

### TRAF7

TRAF7 is a signal transducer within the TNF receptor family and is located on chromosome 16p13. TRAF proteins (TRAF1-7), are adaptor proteins involved in the assembly of intracellular signal transducers downstream of receptor complexes and are composed of an N-terminal RING and zinc finger domain protein. Though TRAF7 was the last in the family of TRAF proteins to be discovered, it appears to involve signal transduction to either activate or suppress signaling through the nuclear factor-kappa-B transcription factor [32]. TRAF7 mutations are present in approximately 25% of sporadic meningiomas, and studies have revealed they are mutually exclusive from NF2 mutant tumors, yet larger studies are need to confirm exclusivity of TRAF7 mutant tumors. TRAF7 mutations also present with mutations in AKT1 or KLF4, especially those KLF4 mutations as a result of the recurrent K409Q mutation [33]. Tumors with mutations in both TRAF7 and KLF4 are most often found in secretory meningiomas with TRAF7 mutant tumors having a predilection for the anterior skull base [24].

### AKT1

The phosphoinositol-3 kinase (PI3K) pathway is a known oncogenic pathway in many cancers including CNS, breast, prostate, urothelial, and ovarian cancers, amongst others. Its downstream targets affect cellular metabolism, motility, proliferation, growth, and survival, making this one of the most frequently dysregulated pathways in human cancers. In brief, activation of PI3K leads to the phosphorylation of AKT1, and subsequent activation of mammalian target of rapamycin (mTOR) [34]. In a large cohort, mutations in AKT1 were identified in 38 of 300 non-irradiated meningioma samples (12.6%) and frequently occurred with TRAF7 mutations (25/38 tumors) [23]. Similarly, in a series of 150 primarily grade 1 meningiomas, mutations in AKT1 occurred in 9% of tumors. The specific mutation, AKT1^E17K^ commonly presents in meningothelial or transitional meningioma histologies [29, 35]. PIK3CA, located on chromosome 3q26.3 encodes the p110 alpha catalytic subunit of PI3K, occurs in 7% of non-NF2 associated grade I meningiomas, and has been reported in grade 2 and grade 3 tumors [36, 37]. Interestingly, AKT1 and PIK3CA are mutually exclusive and, similar to tumors harboring AKT1 mutations, those harboring PIK3CA mutations also harbored TRAF7 mutations, though lacked mutations in NF2 or SMO [22]. In both studies, AKT1 and PIK3CA mutated meningiomas were primarily located along the midline anterior skull base including olfactory groove, tuberculum sellae, anterior clinoid, and medial sphenoid wing. Interestingly, though AKT1 and KLF4 mutations can both occur with TRAF7 mutations, AKT1 and KLF4 mutations are mutually exclusive [22, 29].

### KLF4

KLF4 encodes a zinc finger transcription factor involved in cell growth, proliferation, and differentiation as well as reprogramming differentiated somatic cells into pluripotent stem cells [38, 39]. KLF4 mutations are nearly exclusively found in WHO grade 1 meningiomas and are often found with TRAF7 mutations. Tumors with KLF4 mutations are associated with larger peritumoral brain edema, localize to the anterior and middle cranial skull base, and when present with TRAF7 mutations are predictive of a secretory meningioma phenotype [24, 40].

### SMO

SMO is a gene encoding the 7-transmembrane domain protein, Smoothened, and is part of the sonic hedgehog (SHH) pathway. Activation of the SHH pathway causes a signaling cascade leading to the activation of zinc finger transcription factors which plays a key role in embryogenesis, angiogenesis, proliferation, and survival. Most notably the SHH pathway is involved in the development of the ventral forebrain and midline anterior cranial skull base with abnormalities in signaling causing holoprosencephaly [41]. Activation of the SHH pathway is also implicated in other cancer types including medulloblastoma and basal cell carcinoma. Multiple studies have shown SMO to be an independent mutation causing meningioma oncogenesis with an incidence of 3.6–6% of tested tumors [22–24]. The majority of tumors caused by mutations in SMO were mutually exclusive from other known mutations and gave rise almost exclusively to olfactory groove meningiomas. Of tested olfactory groove meningiomas, approximately 28% harbor a SMO mutation compared to 3–5% at other intracranial locations. In addition, olfactory groove meningiomas harboring a SMO mutation had a higher recurrence rate and shorter progression free survival compared to those olfactory groove meningiomas resulting from mutations in AKT1 [42]. Interestingly, pathway analysis of the known oncogenic downstream marker GRB2-associated-binding protein 1 (GAB-1), which is used to show SHH pathway activation in medulloblastoma, and Stathmin (STMN-1), a downstream marker of the PI3K/AKT/mToR pathway, were positive in three SMO-mutant tumors, suggesting a possible interaction in these parallel oncogenic pathways [23].

### BAP1

Breast cancer type 1 susceptibility protein associated protein-1 (BAP1) is a tumor suppressor gene and deubiquitylase that regulates multiple cellular pathways including cell cycle progression, gluconeogenesis, and the DNA damage response. Germline BAP1 mutations cause a tumor syndrome composed of mesothelioma, cutaneous and uveal melanomas, meningioma, and renal cell carcinoma, amongst others [43]. BAP1 mutations are specifically associated with meningiomas with rhabdoid morphology and can occur in both the adult and pediatric population [44]. Shankar reported in a series of 14 meningiomas with rhabdoid features, that loss of BAP1 resulted in decreased progression free survival compared to those tumors without BAP1 mutations [25].

### POLR2A

POLR2A, present on chromosome 17 encodes for the largest subunit of the ubiquitous protein, RNA polymerase II, which is responsible for synthesizing messenger RNA in eukaryotes. Mutations in POLR2A lead to dysregulation of key meningeal identity genes ranging from wingless-type MMTV integration site family, member 6 (WNT6) to alpha prolamins (ZIC1) and are found in approximately 6% of meningiomas. POLR2A- mutant meningiomas are WHO grade 1, are more likely present with a meningothelial histology, and are commonly located in the anterior cranial skull base [27].

### SMARCB1

SMARCB1 is a tumor suppressor gene involved in chromatin remodeling and is a key component of the Switch/sucrose non-fermentable chromatin-remodeling complex protein. Mutations in SMARCB1 have been identified in a rarity of sporadic WHO grade 1 and 2 meningiomas, often colocalizing with NF2 mutations. Interestingly, germline SMARCB1 mutations appear to also be involved in familial cases of multiple meningiomas and schwannomas [45, 46]. SMARCB1 mutant tumors have a predilection for developing along the falx cerebri [47].

### Proteomic markers

While the genomic landscape of meningiomas has provided insight into the molecular biology and drivers of clinical course, the protein-level understanding of meningiomas remains largely unexplored. Proteomic markers along with genomics will aid in the development of a real-time clinical biomarker. Imploring global shotgun level proteomics has highlighted proteins and pathways pertinent to the clinical course of meningiomas [48–50]. In examining 61 meningiomas and quantifying 3042 unique proteins, distinct patterns are observed between benign and atypical grades with oncogenes being enriched in higher grades [51]. Differences across WHO grades are seen in RNA metabolism, extracellular matrix formation, mitochondrial metabolism with grade 1 tumors exhibiting enrichment in the matrisome and the biosynthesis of glycosaminoglycans [51]. Proteomic analysis has identified downstream protein-level changes in meningiomas based on their spatial distributions [51]. Shotgun proteomics has also demonstrated differences in grade 1 meningiomas presenting in different genders. Differences in RNA splicing events (S100-A4) were observed between the two groups, with males exhibiting enriched pathways for cell-matrix organization and females exhibiting enriched pathways for RNA transport and processing [52]. Further proteomics studies have been able to narrow down which proteins may serve as a potential therapeutic target. In an examination of NF2 mutated meningiomas, upregulated proteins combined either a PDZ/LIM domain which play a wide role in biological functions, specifically cell signaling [53]. Furthermore, in expanding on these domains, PDZ and LIM domain protein 2 (PDLIM2/mystique/SLIM) was found to be dysregulated with overexpression in several tumor samples. Knockdown of PDLIM2 mediated by shRNA resulted in significant reductions in cellular proliferation [53]. Additional proteomic studies assessed for biomarkers in serum, cerebrospinal fluid, and pathological tissue and identified multiple proteins related to tumorigenesis and meningioma grading. These included serpin peptidase inhibitor alpha 1, ceruloplasmin, hemopexin, albumin, C3, apolipoprotein, haptoglobin, amyloid P-component serum, alpha-1-beta-glycoprotein, alpha-2-macroglobulin, and antithrombin-III [54, 55]. These proteins and their associated pathways may serve as potential therapeutic targets or can help further differentiate which meningiomas demonstrate higher rates of progression/recurrence.

**Figure 2:**
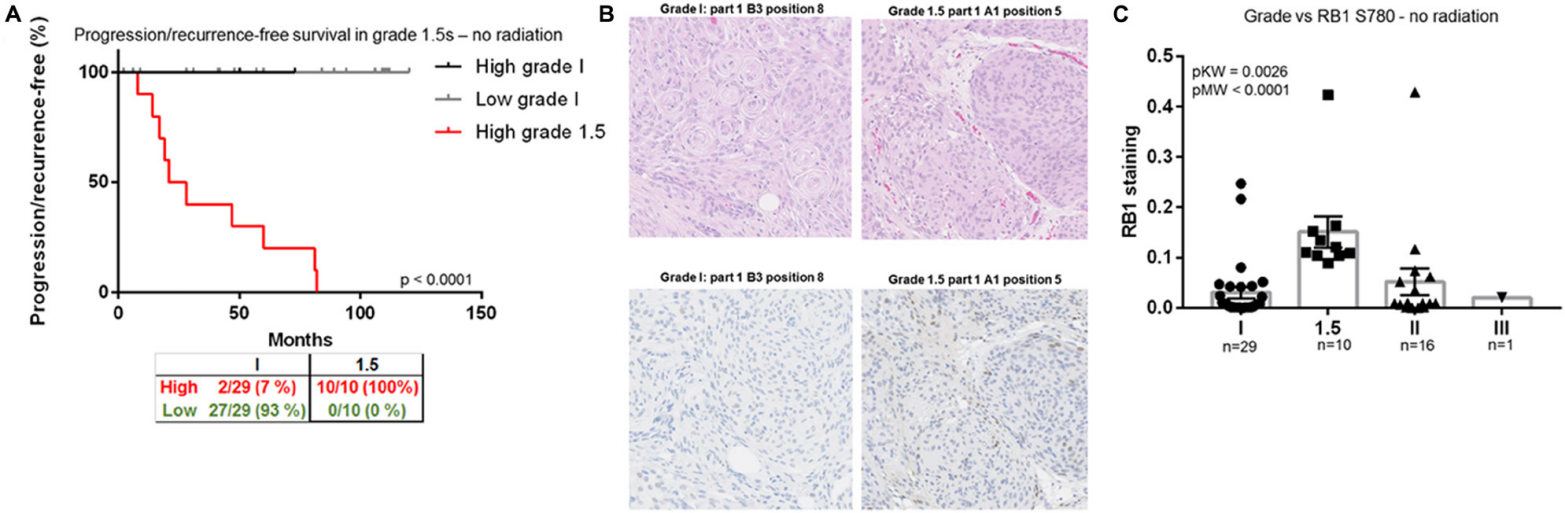
RB1, a biomarker for identification of grade 1.5 meningiomas. (**A**) Progression/recurrence-free survival in all nonirradiated histologically benign meningiomas (grades 1, *n* = 47, and 1.5, *n* = 28). (**B**) Representative WHO 1 and 1.5 meningioma H&E section from two cases. The same representative cases stained with RB1 S780. Note the increased cytoplasmic and nuclear staining in the 1.5 meningioma. (**C**) Ratio of RB1 S780 staining per total tissue area versus tumor grade–nonirradiated samples only. These figures were reproduced with permission by the publisher of the CCR manuscript.

To this point, our group used a proteomic approach, identifying retinoblastoma associated protein-1 (RB1) as a critical marker to identify grade 1 tumors at high risk for recurrence. These tumors that progressed or recurred within five years, are referred to as grade 1.5. RB1 regulates cell cycle progression and drives the progression from the G1 to S-phase of the cell cycle. Using mass spectrometry-based phosphoproteomics, peptide chip array kinomics, and clinical outcomes we compared a cohort of 140 meningiomas grade 1 and 1.5 tumors. Multiple sites on the RB1 protein can be phosphorylated and through kinome profiling, we identified Rb1 pS780 to be a sensitive biomarker for identification of non-irradiated grade 1 tumors that showed early recurrence or progression (Figure 2A). Histologically, there was no difference between grade 1 and grade 1.5 tumors, though RB1 S780 was significantly higher in non-irradiated grade 1.5 tumors compared to either non-irradiated traditional WHO grade 1, 2, and 3 tumors. Staining of RB1 S780 is also found in half of the WHO grade 2 and 3 meningiomas displaying aggressive invasion and spread (Figure 2B and 2C). Genomic sequencing was also performed and mutations in NF2, TRAF7, SMO, KLF4, and AKT1 did not predict RB1 S780 staining or progression in grade 1.5 meningiomas [56].

We hypothesize that RB1 S780 hyperphosphorylation is the result of genetic and/or epigenetic alterations and an early event in aggressive progression in meningiomas. Unlike the genomic underpinnings which have led to a revolution in identifying cellular signaling pathways involved in tumorigenesis, RB1 S780 provides a biomarker to better understand which patients may require closure follow-up or consideration of adjuvant therapies.

### Epigenetic and mRNA markers

Genotyping only partially explains the early events of tumorigenesis, and there is evidence that epigenetic abnormalities are crucial for tumorigenesis. A combination of epigenetics, mRNA along with genomics and proteomics will allow for a meningioma biomarker panel to predict aggressive meningiomas exhibiting high rates of progression or recurrence (Figure 1). Abnormalities ranging from altered DNA methylation, miRNA expression and chromatin restructuring involved in the genesis of meningiomas still remains unclear. One such example is tissue inhibitor of metalloproteinase 3 (TIMP3), which encodes a protein that inhibits matrix metalloproteinase. It has been demonstrated that hypermethylation results in transcriptional downregulation with Grade 1 tumors displaying less methylation than Grade 2 and 3 meningiomas [57, 58]. Additional tumor suppressor proteins such as p73 and p53 are known to play a role in meningiomas in which hypermethylation and expression characterizes low- and high-grade meningiomas [59, 60]. Genes with hypermethylated CpG islands in promoter regions are suppressed in benign and malignant meningiomas indicating that gene silencing is induced by DNA methylation.

More recently, global methylation profiles have shown promise in identifying those tumors at high risk for recurrence. Using unsupervised clustering of DNA methylation, Olar and colleagues reported a 64-CpG loci methylation predictor, that, when controlled for clinical variables including WHO grade, mitotic index, Simpson grade, sex, location, and copy number aberrations, showed a significant association with recurrence free survival [60]. Using similar methods, six unique methylation classes of meningioma with accurate identification of WHO grade 1 at high risk of recurrence and WHO grade 2 tumors at lower risk of recurrence were identified [61].

Another form of genetic modification is chromatin restructuring in which trinucleotide repeat expansions affect the neural system but has not directly been implicated in meningiomas [62]. Microsatellite instability was reported and occurred in 11% of meningiomas [62]. The final set of epigenetic abnormalities are miRNAs that play regulatory roles in cell cycle, differentiation, migration and apoptosis. Various levels of miRNAs were reported in higher grade meningiomas compared to lower grades [63]. MiRNA expression profiling revealed high and low expression of a variety of miRNAs which are linked to higher recurrence rates of meningiomas [64]. Downregulation of miR-200a plays a pivotal role in the development of benign meningiomas through the upregulation of three mRNA targets while miR-21 and 34a have represented barriers for grades 1 and 2 tumors to malignant transition [65, 66]. MiRNA expression in meningiomas is varied across grades and especially plays a key role in the early stages of tumorigenesis and may provide a potential therapeutic target in the future.

### Translational potential for systemic therapy

Currently, the gold standard for meningioma treatment involves maximal safe surgical resection for those tumors causing neurologic compromise or significant mass effect. The evolution of meningioma surgery has been significantly refined since Simpson’s landmark paper showing improved progression free survival with gross total resection of the tumor bulk and involved dura [10]. Introduction of the operating microscope and advancements in neuro-endoscopy dramatically increases illumination and magnification of deep seated structures [67, 68]. In combination with fine micro-instruments, tumors can be dissected from cranial nerves and vasculature and major dural sinuses can be entered and reconstructed, limiting the morbidity of surgery. Advancements in surgical neuromonitoring can detect ischemic or pressure related changes in cranial nerves and brain parenchyma to assist the surgeon in safe resection [69]. Finally, advancements in skull base approaches and intra-operative neuro-navigation allow for safe access to deep seated tumors with minimal brain retraction or sectioning cranial nerves for safe resection.

With the advancements in surgical technique weighed against the overall spectrum of responsiveness to radiation, the pendulum has swung towards aggressive surgical resection with radiation of any kind reserved for higher grade meningiomas or growing residual tumors that could not be safely resected. In this setting, an effective medical therapy would provide a greatly needed clinical alternative to radiation. Unfortunately, no randomized, double-blind, placebo controlled clinical trial has identified a cytotoxic chemo, hormonal, biologic, or immunotherapy that provides durable and reproducible tumor control. While multifactorial, this is likely due to the overall indolent nature of the majority of meningiomas, requiring a balance in studying progression and survival outcomes with the expense of years or decades long clinical and radiographic follow-up, limited cohorts that would sufficiently power a randomized study, and non-standardized clinical metrics making comparison difficult. Results of chemo, biologic, and immunotherapies in meningioma are reviewed elsewhere.

Despite these challenges, advancements in meningioma genomics and proteomics has provided significant insight for possible targeted therapies and identifying tumors at high risk of recurrence. Specifically, anecdotal evidence suggests that inhibitors to AKT1 may provide clinical benefit. In a single patient with multiple multi-treatment resistant meningiomas including grade 1 and grade 3 intracranial disease and pulmonary metastases, treatment with the AKT inhibitor AZD5363 led to radiographic response of both intracranial and pulmonary lesions with a durable response over 18 months of follow-up [70]. Currently, clinical trials of AZD5363 are ongoing for meningioma as well as other cancer types [71]. Currently, clinical trials evaluating the efficacy of SMO inhibitors are also enrolling [72]. Evidence from prior trials testing the SMO inhibitor, vismodegib, in basal cell carcinoma showed excellent local and distant control, leading to its FDA approval in 2012 [73]. While no current trials are ongoing with specific inhibitors of BAP1 in meningioma, Tazemetostat, an inhibitor of enhancer of zeste homolog-2 which is a downstream protein of BAP1, is currently being evaluated in mesothelioma, and may represent a potential targeted therapeutic for rhabdoid meningioma. Finally, genomic markers can be used to assist in screening for extracranial sites of other potential tumors (BAP1 tumor syndrome) and familial syndromes of multiple meningiomas (SMARCB1). In addition, proteomic findings such as RB1 S780 and epigenetic modifications may better classify tumors at high risk of recurrence as compared to the current WHO grading system.

## CONCLUSIONS

As further advancements in targeted therapies arise, the role of surgery will expand to not only include reduction of tumor mass but also providing tumor sample for identification of actionable mutations and identification of prognostic markers to better predict prognosis. In addition, identification of specific mutations can aid in further screening of tumor syndromes. Due to the heterogenous nature of meningiomas a combination of their genomic, proteomic, and epigenetic landscapes will form the basis for meningioma diagnosis, prognosis, and treatment.

## References

[1] Ostrom QT , Gittleman H , Truitt G , Boscia A , Kruchko C , Barnholtz-Sloan JS . CBTRUS Statistical Report: Primary Brain and Other Central Nervous System Tumors Diagnosed in the United States in 2011–2015. Neuro Oncol. 2018; 20:iv1–iv86. 10.1093/neuonc/noy131. 30445539 PMC6129949

[2] Louis DN , Perry A , Reifenberger G , von Deimling A , Figarella-Branger D , Cavenee WK , Ohgaki H , Wiestler OD , Kleihues P , Ellison DW . The 2016 World Health Organization Classification of Tumors of the Central Nervous System: a summary. Acta Neuropathol. 2016; 131:803–820. 10.1007/s00401-016-1545-1. 27157931

[3] Nakasu S , Fukami T , Jito J , Nozaki K . Recurrence and regrowth of benign meningiomas. Brain Tumor Pathol. 2009; 26:69–72. 10.1007/s10014-009-0251-2. 19856217

[4] Marciscano AE , Stemmer-Rachamimov AO , Niemierko A , Larvie M , Curry WT , Barker FG , Martuza RL , McGuone D , Oh KS , Loeffler JS , Shih HA . Benign meningiomas (WHO Grade I) with atypical histological features: correlation of histopathological features with clinical outcomes. J Neurosurg. 2016; 124:106–114. 10.3171/2015.1.JNS142228. 26274991

[5] Aizer AA , Bi WL , Kandola MS , Lee EQ , Nayak L , Rinne ML , Norden AD , Beroukhim R , Reardon DA , Wen PY , Al-Mefty O , Arvold ND , Dunn IF , et al. Extent of resection and overall survival for patients with atypical and malignant meningioma. Cancer. 2015; 121:4376–4381. 10.1002/cncr.29639. 26308667

[6] Cao X , Hao S , Wu Z , Wang L , Jia G , Zhang L , Zhang J . Survival rates, prognostic factors and treatment of anaplastic meningiomas. J Clin Neurosci. 2015; 22:828–833. 10.1016/j.jocn.2014.11.022. 25827863

[7] Moliterno J , Cope WP , Vartanian ED , Reiner AS , Kellen R , Ogilvie SQ , Huse JT , Gutin PH . Survival in patients treated for anaplastic meningioma. J Neurosurg. 2015; 123:23–30. 10.3171/2014.10.JNS14502. 25859807

[8] Rosenberg LA , Prayson RA , Lee J , Reddy C , Chao ST , Barnett GH , Vogelbaum MA , Suh JH . Long-Term Experience With World Health Organization Grade III (Malignant) Meningiomas at a Single Institution. Int J Radiat Oncol Biol Phys. 2009; 74:427–432. 10.1016/j.ijrobp.2008.08.018. 19427553

[9] Sughrue ME , Sanai N , Shangari G , Parsa AT , Berger MS , McDermott MW . Outcome and survival following primary and repeat surgery for World Health Organization Grade III meningiomas. J Neurosurg. 2010; 113:202–209. 10.3171/2010.1.JNS091114. 20225922

[10] Simpson D . The recurrence of intracranial meningiomas after surgical treatment. J Neurol Neurosurg Psychiatry. 1957; 20:22–39. 10.1136/jnnp.20.1.22. 13406590 PMC497230

[11] Nanda A , Bir SC , Maiti TK , Konar SK , Missios S , Guthikonda B . Relevance of Simpson grading system and recurrence-free survival after surgery for World Health Organization Grade I meningioma. J Neurosurg. 2017; 126:201–211. 10.3171/2016.1.JNS151842. 27058201

[12] Gousias K , Schramm J , Simon M . The Simpson grading revisited: aggressive surgery and its place in modern meningioma management. J Neurosurg. 2016; 125:551–560. 10.3171/2015.9.JNS15754. 26824369

[13] Li H , Zhang YS , Zhang GB , Zhang GJ , Wang B , Li D , Wu Z , Zhang JT . Treatment Protocol, Long-Term Follow-Up, and Predictors of Mortality in 302 Cases of Atypical Meningioma. World Neurosurg. 2019; 122:e1275–e1284. 10.1016/j.wneu.2018.11.032. 30447439

[14] Valery CA , Faillot M , Lamproglou I , Golmard JL , Jenny C , Peyre M , Mokhtari K , Mazeron JJ , Cornu P , Kalamarides M . Grade II meningiomas and Gamma Knife radiosurgery: analysis of success and failure to improve treatment paradigm. J Neurosurg. 2016; 125:89–96. 10.3171/2016.7.GKS161521. 27903189

[15] Sun SQ , Kim AH , Cai C , Murphy RKJ , DeWees T , Sylvester P , Dacey RG , Grubb RL , Rich KM , Zipfel GJ , Dowling JL , Leuthardt EC , Leonard JR , et al. Management of atypical cranial meningiomas, part 1: predictors of recurrence and the role of adjuvant radiation after gross total resection. Neurosurgery. 2014; 75:347–354. 10.1227/NEU.0000000000000461. 24932707

[16] Sun SQ , Cai C , Murphy RKJ , DeWees T , Dacey RG , Grubb RL , Rich KM , Zipfel GJ , Dowling JL , Leuthardt EC , Leonard JR , Evans J , Simpson JR , et al. Management of atypical cranial meningiomas, part 2: predictors of progression and the role of adjuvant radiation after subtotal resection. Neurosurgery. 2014; 75:356–363, discussion 363. 10.1227/NEU.0000000000000462. 24932708

[17] Gabeau-Lacet D , Aghi M , Betensky RA , Barker FG , Loeffler JS , Louis DN . Bone involvement predicts poor outcome in atypical meningioma. J Neurosurg. 2009; 111:464–471. 10.3171/2009.2.JNS08877. 19267533 PMC2845926

[18] Palma L , Celli P , Franco C , Cervoni L , Cantore G . Long-term prognosis for atypical and malignant meningiomas: a study of 71 surgical cases. J Neurosurg. 1997; 86:793–800. 10.3171/jns.1997.86.5.0793. 9126894

[19] Aghi MK , Carter BS , Cosgrove GR , Ojemann RG , Amin-Hanjani S , Martuza RL , Curry WT , Barker FG . Long-term recurrence rates of atypical meningiomas after gross total resection with or without postoperative adjuvant radiation. Neurosurgery. 2009; 64:56–60. 10.1227/01.NEU.0000330399.55586.63. 19145156

[20] Parsons DW , Jones S , Zhang X , Lin JC , Leary RJ , Angenendt P , Mankoo P , Carter H , Siu IM , Gallia GL , Olivi A , McLendon R , Rasheed BA , et al. An Integrated Genomic Analysis of Human Glioblastoma Multiforme. Science. 2008; 321:1807–1812. 10.1126/science.1164382. 18772396 PMC2820389

[21] Hegi ME , Diserens AC , Gorlia T , Hamou MF , de Tribolet N , Weller M , Kros JM , Hainfellner JA , Mason W , Mariani L , Bromberg JEC , Hau P , Mirimanoff RO , et al. MGMT Gene Silencing and Benefit from Temozolomide in Glioblastoma. N Engl J Med. 2005; 352:997–1003. 10.1056/NEJMoa043331. 15758010

[22] Abedalthagafi M , Bi WL , Aizer AA , Merrill PH , Brewster R , Agarwalla PK , Listewnik ML , Dias-Santagata D , Thorner AR , Van Hummelen P , Brastianos PK , Reardon DA , Wen PY , et al. Oncogenic PI3K mutations are as common as AKT1 and SMO mutations in meningioma. Neuro Oncol. 2016; 18:649–655. 10.1093/neuonc/nov316. 26826201 PMC4827048

[23] Brastianos PK , Horowitz PM , Santagata S , Jones RT , McKenna A , Getz G , Ligon KL , Palescandolo E , Van Hummelen P , Ducar MD , Raza A , Sunkavalli A , Macconaill LE , et al. Genomic sequencing of meningiomas identifies oncogenic SMO and AKT1 mutations. Nat Genet. 2013; 45:285–289. 10.1038/ng.2526. 23334667 PMC3739288

[24] Clark VE , Erson-Omay EZ , Serin A , Yin J , Cotney J , Ozduman K , Avsar T , Li J , Murray PB , Henegariu O , Yilmaz S , Gunel JM , Carrion-Grant G , et al. Genomic Analysis of Non-NF2 Meningiomas Reveals Mutations in TRAF7, KLF4, AKT1, and SMO. Science. 2013; 339:1077–1080. 10.1126/science.1233009. 23348505 PMC4808587

[25] Shankar GM , Abedalthagafi M , Vaubel RA , Merrill PH , Nayyar N , Gill CM , Brewster R , Bi WL , Agarwalla PK , Thorner AR , Reardon DA , Al-Mefty O , Wen PY , et al. Germline and somatic BAP1 mutations in high-grade rhabdoid meningiomas. Neuro Oncol. 2017; 19:535–545. 10.1093/neuonc/nox094. 28170043 PMC5464371

[26] Karsy M , Azab MA , Abou-Al-Shaar H , Guan J , Eli I , Jensen RL , Ormond DR . Clinical potential of meningioma genomic insights: a practical review for neurosurgeons. Neurosurg Focus. 2018; 44:E10. 10.3171/2018.2.FOCUS1849. 29852774

[27] Clark VE , Harmancı AS , Bai H , Youngblood MW , Lee TI , Baranoski JF , Ercan-Sencicek AG , Abraham BJ , Weintraub AS , Hnisz D , Simon M , Krischek B , Erson-Omay EZ , et al. Recurrent somatic mutations in POLR2A define a distinct subset of meningiomas. Nat Genet. 2016; 48:1253–1259. 10.1038/ng.3651. 27548314 PMC5114141

[28] Harvey KF , Zhang X , Thomas DM . The Hippo pathway and human cancer. Nat Rev Cancer. 2013; 13:246–257. 10.1038/nrc3458. 23467301

[29] Yuzawa S , Nishihara H , Tanaka S . Genetic landscape of meningioma. Brain Tumor Pathol. 2016; 33:237–247. 10.1007/s10014-016-0271-7. 27624470

[30] Smith MJ . Germline and somatic mutations in meningiomas. Cancer Genet. 2015; 208:107–114. 10.1016/j.cancergen.2015.02.003. 25857641

[31] Lee S , Karas PJ , Hadley CC , Bayley V JC , Khan AB , Jalali A , Sweeney AD , Klisch TJ , Patel AJ . The Role of Merlin/NF2 Loss in Meningioma Biology. Cancers (Basel). 2019; 11:1633. 10.3390/cancers11111633. 31652973 PMC6893739

[32] Zotti T , Vito P , Stilo R . The seventh ring: exploring TRAF7 functions. J Cell Physiol. 2012; 227:1280–1284. 10.1002/jcp.24011. 22105767

[33] von Spreckelsen N , Waldt N , Poetschke R , Kesseler C , Dohmen H , Jiao HK , Nemeth A , Schob S , Scherlach C , Sandalcioglu IE , Deckert M , Angenstein F , Krischek B , et al. KLF4K409Q–mutated meningiomas show enhanced hypoxia signaling and respond to mTORC1 inhibitor treatment. Acta Neuropathol Commun. 2020; 8:41. 10.1186/s40478-020-00912-x. 32245394 PMC7118946

[34] Janku F , Yap TA , Meric-Bernstam F . Targeting the PI3K pathway in cancer: are we making headway? Nat Rev Clin Oncol. 2018; 15:273–291. 10.1038/nrclinonc.2018.28. 29508857

[35] Sahm F , Bissel J , Koelsche C , Schweizer L , Capper D , Reuss D , Böhmer K , Lass U , Göck T , Kalis K , Meyer J , Habel A , Brehmer S , et al. AKT1E17K mutations cluster with meningothelial and transitional meningiomas and can be detected by SFRP1 immunohistochemistry. Acta Neuropathol. 2013; 126:757–762. 10.1007/s00401-013-1187-5. 24096618

[36] Bujko M , Kober P , Tysarowski A , Matyja E , Mandat T , Bonicki W , Siedlecki JA . EGFR, PIK3CA, KRAS and BRAF mutations in meningiomas. Oncol Lett. 2014; 7:2019–2022. 10.3892/ol.2014.2042. 24932282 PMC4049666

[37] Pang JC , Chung NY , Chan NH , Poon WS , Thomas T , Ng HK . Rare mutation of PIK3CA in meningiomas. Acta Neuropathol. 2006; 111:284–285. 10.1007/s00401-005-0021-0. 16463202

[38] Takahashi K , Tanabe K , Ohnuki M , Narita M , Ichisaka T , Tomoda K , Yamanaka S . Induction of pluripotent stem cells from adult human fibroblasts by defined factors. Cell. 2007; 131:861–872. 10.1016/j.cell.2007.11.019. 18035408

[39] Ghaleb AM , Yang VW . Krüppel-like factor 4 (KLF4): What we currently know. Gene. 2017; 611:27–37. 10.1016/j.gene.2017.02.025. 28237823 PMC5391259

[40] Youngblood MW , Duran D , Montejo JD , Li C , Omay SB , Ö zduman K , Sheth AH , Zhao AY , Tyrtova E , Miyagishima DF , Fomchenko EI , Hong CS , Clark VE , et al. Correlations between genomic subgroup and clinical features in a cohort of more than 3000 meningiomas. J Neurosurg. 2020; 133:1285–1633. 10.3171/2019.8.JNS191266. 31653806

[41] Xavier GM , Seppala M , Barrell W , Birjandi AA , Geoghegan F , Cobourne MT . Hedgehog receptor function during craniofacial development. Dev Biol. 2016; 415:198–215. 10.1016/j.ydbio.2016.02.009. 26875496

[42] Boetto J , Bielle F , Sanson M , Peyre M , Kalamarides M . SMO mutation status defines a distinct and frequent molecular subgroup in olfactory groove meningiomas. Neuro Oncol. 2017; 19:345–351. 10.1093/neuonc/now276. 28082415 PMC5464306

[43] Carbone M , Yang H , Pass HI , Krausz T , Testa JR , Gaudino G . BAP1 and cancer. Nat Rev Cancer. 2013; 13:153–159. 10.1038/nrc3459. 23550303 PMC3792854

[44] Ravanpay AC , Barkley A , White-Dzuro GA , Cimino PJ , Gonzalez-Cuyar LF , Lockwood C , Halasz LM , Hisama FM , Ferreira M . Giant Pediatric Rhabdoid Meningioma Associated with a Germline BAP1 Pathogenic Variation: A Rare Clinical Case. World Neurosurg. 2018; 119:402–415. 10.1016/j.wneu.2018.06.227. 29981911

[45] Christiaans I , Kenter SB , Brink HC , van Os TA , Baas F , van den Munckhof P , Kidd AM , Hulsebos TJ . Germline SMARCB1 mutation and somatic NF2 mutations in familial multiple meningiomas. J Med Genet. 2011; 48:93–97. 10.1136/jmg.2010.082420. 20930055

[46] Bacci C , Sestini R , Provenzano A , Paganini I , Mancini I , Porfirio B , Vivarelli R , Genuardi M , Papi L . Schwannomatosis associated with multiple meningiomas due to a familial SMARCB1 mutation. Neurogenetics. 2010; 11:73–80. 10.1007/s10048-009-0204-2. 19582488

[47] van den Munckhof P , Christiaans I , Kenter SB , Baas F , Hulsebos TJ . Germline SMARCB1 mutation predisposes to multiple meningiomas and schwannomas with preferential location of cranial meningiomas at the falx cerebri. Neurogenetics. 2012; 13:1–7. 10.1007/s10048-011-0300-y. 22038540

[48] Okamoto H , Li J , Vortmeyer AO , Jaffe H , Lee YS , Gläsker S , Sohn TS , Zeng W , Ikejiri B , Proescholdt MA , Mayer C , Weil RJ , Oldfield EH , et al. Comparative Proteomic Profiles of Meningioma Subtypes. Cancer Res. 2006; 66:10199–10204. 10.1158/0008-5472.CAN-06-0955. 17047085

[49] Sharma S , Ray S , Mukherjee S , Moiyadi A , Sridhar E , Srivastava S . Multipronged quantitative proteomic analyses indicate modulation of various signal transduction pathways in human meningiomas. Proteomics. 2015; 15:394–407. 10.1002/pmic.201400328. 25413884

[50] Barkhoudarian G , Whitelegge JP , Kelly DF , Simonian M . Proteomics Analysis of Brain Meningiomas in Pursuit of Novel Biomarkers of the Aggressive Behavior. J Proteomics Bioinform. 2016; 9:53–57. 10.4172/jpb.1000389. 27019568 PMC4807620

[51] Papaioannou MD , Djuric U , Kao J , Karimi S , Zadeh G , Aldape K , Diamandis P . Proteomic analysis of meningiomas reveals clinically-distinct molecular patterns. Neuro Oncol. 2019; 21:1028–1038. 10.1093/neuonc/noz084. 31077268 PMC6682208

[52] Silva JM , Wippel HH , Santos MDM , Verissimo DCA , Santos RM , Nogueira FCS , Passos GAR , Sprengel SL , Borba LAB , Carvalho PC , Fischer JSDG . Proteomics pinpoints alterations in grade I meningiomas of male versus female patients. Sci Rep. 2020; 10:10355. 10.1038/s41598-020-67113-3. 32587372 PMC7316823

[53] Bassiri K , Ferluga S , Sharma V , Syed N , Adams CL , Lasonder E , Hanemann CO . Global Proteome and Phospho-proteome Analysis of Merlin-deficient Meningioma and Schwannoma Identifies PDLIM2 as a Novel Therapeutic Target. EBioMedicine. 2017; 16:76–86. 10.1016/j.ebiom.2017.01.020. 28126595 PMC5474504

[54] Abbritti RV , Polito F , Cucinotta M , Lo Giudice C , Caffo M , Tomasello C , Germanò A , Aguennouz M . Meningiomas and Proteomics: Focus on New Potential Biomarkers and Molecular Pathways. Cancer Genomics Proteomics. 2016; 13:369–379. 27566655 PMC5070626

[55] Sharma S , Ray S , Moiyadi A , Sridhar E , Srivastava S . Quantitative Proteomic Analysis of Meningiomas for the Identification of Surrogate Protein Markers. Sci Rep. 2014; 4:7140. 10.1038/srep07140. 25413266 PMC5382771

[56] Parada CA , Osbun JW , Busald T , Karasozen Y , Kaur S , Shi M , Barber J , Adidharma W , Cimino PJ , Pan C , Gonzalez-Cuyar LF , Rostomily R , Born DE , et al. Phosphoproteomic and Kinomic Signature of Clinically Aggressive Grade I (1.5) Meningiomas Reveals RB1 Signaling as a Novel Mediator and Biomarker. Clin Cancer Res. 2020; 26:193–205. 10.1158/1078-0432.CCR-18-0641. 31615938

[57] Barski D , Wolter M , Reifenberger G , Riemenschneider MJ . Hypermethylation and transcriptional downregulation of the TIMP3 gene is associated with allelic loss on 22q12.3 and malignancy in meningiomas. Brain Pathol. 2010; 20:623–631. 10.1111/j.1750-3639.2009.00340.x. 19922547 PMC8094659

[58] Linsler S , Kraemer D , Driess C , Oertel J , Kammers K , Rahnenführer J , Ketter R , Urbschat S . Molecular biological determinations of meningioma progression and recurrence. PLoS One. 2014; 9:e94987. 10.1371/journal.pone.0094987. 24722350 PMC3983248

[59] Murnyák B , Bognár L , Klekner Á , Hortobágyi T . Epigenetics of Meningiomas. Biomed Res Int. 2015; 2015:532451. 10.1155/2015/532451. 26101774 PMC4458517

[60] Olar A , Wani KM , Wilson CD , Zadeh G , DeMonte F , Jones DTW , Pfister SM , Sulman EP , Aldape KD . Global epigenetic profiling identifies methylation subgroups associated with recurrence-free survival in meningioma. Acta Neuropathol. 2017; 133:431–444. 10.1007/s00401-017-1678-x. 28130639 PMC5600514

[61] Sahm F , Schrimpf D , Stichel D , Jones DTW , Hielscher T , Schefzyk S , Okonechnikov K , Koelsche C , Reuss DE , Capper D , Sturm D , Wirsching HG , Berghoff AS , et al. DNA methylation-based classification and grading system for meningioma: a multicentre, retrospective analysis. Lancet Oncol. 2017; 18:682–694. 10.1016/S1470-2045(17)30155-9. 28314689

[62] Bi WL , Greenwald NF , Abedalthagafi M , Wala J , Gibson WJ , Agarwalla PK , Horowitz P , Schumacher SE , Esaulova E , Mei Y , Chevalier A , A. Ducar M , Thorner AR , et al. Genomic landscape of high-grade meningiomas. NPJ Genom Med. 2017; 2:15. 10.1038/s41525-017-0014-7. 28713588 PMC5506858

[63] Zhi F , Shao N , Li B , Xue L , Deng D , Xu Y , Lan Q , Peng Y , Yang Y . A serum 6-miRNA panel as a novel non-invasive biomarker for meningioma. Sci Rep. 2016; 6:32067. 10.1038/srep32067. 27558167 PMC4997338

[64] Zhi F , Zhou G , Wang S , Shi Y , Peng Y , Shao N , Guan W , Qu H , Zhang Y , Wang Q , Yang C , Wang R , Wu S , et al. A microRNA expression signature predicts meningioma recurrence. Int J Cancer. 2013; 132:128–136. 10.1002/ijc.27658. 22674195

[65] Galani V , Lampri E , Varouktsi A , Alexiou G , Mitselou A , Kyritsis AP . Genetic and epigenetic alterations in meningiomas. Clin Neurol Neurosurg. 2017; 158:119–125. 10.1016/j.clineuro.2017.05.002. 28527972

[66] Katar S , Baran O , Evran S , Cevik S , Akkaya E , Baran G , Antar V , Hanimoglu H , Kaynar MY . Expression of miRNA-21, miRNA-107, miRNA-137 and miRNA-29b in meningioma. Clin Neurol Neurosurg. 2017; 156:66–70. 10.1016/j.clineuro.2017.03.016. 28349893

[67] Hardavella G , Ianovici N . Current trends in minimally invasive neurosurgery: neuro-endoscopy. Rev Med Chir Soc Med Nat Iasi. 2005; 109:528–531. 16607744

[68] Hernesniemi J , Niemelä M , Dashti R , Karatas A , Kivipelto L , Ishii K , Rinne J , Ronkainen A , Peláez JG , Koivisto T , Kivisaari R , Shen H , Lehecka M , et al. Principles of microneurosurgery for safe and fast surgery. Surg Technol Int. 2006; 15:305–310. 17029189

[69] Bailo M , Gagliardi F , Boari N , Castellano A , Spina A , Mortini P . The Role of Surgery in Meningiomas. Curr Treat Options Neurol. 2019; 21:51. 10.1007/s11940-019-0587-9. 31560106

[70] Weller M , Roth P , Sahm F , Burghardt I , Schuknecht B , Rushing EJ , Regli L , Lindemann JP , von Deimling A . Durable Control of Metastatic AKT1-Mutant WHO Grade 1 Meningothelial Meningioma by the AKT Inhibitor, AZD5363. J Natl Cancer Inst. 2017; 109:1–4. 10.1093/jnci/djw320. 28376212

[71] National Cancer Institute. Olaparib with and without AZD1775, AZD5363, and AZD2014 in Treating Patients with Advanced Solid Tumors. https://www.cancer.gov/about-cancer/treatment/clinical-trials/search/v?id=NCI-2016-00922&r=1.

[72] National Cancer Institute. Vismodegib, FAK Inhibitor GSK2256098, Capivasertib, and Abemaciclib in Treating Patients with Progressive Meningiomas. https://www.cancer.gov/about-cancer/treatment/clinical-trials/search/v?id=NCI-2015-00546&r=1.

[73] Sekulic A , Migden MR , Basset-Seguin N , Garbe C , Gesierich A , Lao CD , Miller C , Mortier L , Murrell DF , Hamid O , Quevedo JF , Hou J , McKenna E , et al. Long-term safety and efficacy of vismodegib in patients with advanced basal cell carcinoma: final update of the pivotal ERIVANCE BCC study. BMC Cancer. 2017; 17:332. 10.1186/s12885-017-3286-5. 28511673 PMC5433030

